# A Comparison of Myoelectric Control Modes for an Assistive Robotic Virtual Platform

**DOI:** 10.3390/bioengineering11050473

**Published:** 2024-05-09

**Authors:** Cristina Polo-Hortigüela, Miriam Maximo, Carlos A. Jara, Jose L. Ramon, Gabriel J. Garcia, Andres Ubeda

**Affiliations:** 1 Brain-Machine Interface Systems Lab, Miguel Hernández University of Elche, 03202 Elche, Spain; cpolo@umh.es; 2 Engineering Research Institute of Elche—I3E, Miguel Hernández University of Elche, 03202 Elche, Spain; mmaximo@umh.es; 3 Human Robotics Group, University of Alicante, 03690 Alicante, Spain; carlos.jara@ua.es (C.A.J.); jl.ramon@ua.es (J.L.R.); gjgg@ua.es (G.J.G.)

**Keywords:** myoelectric control, virtual environments, prosthetic training, activities of daily life

## Abstract

In this paper, we propose a daily living situation where objects in a kitchen can be grasped and stored in specific containers using a virtual robot arm operated by different myoelectric control modes. The main goal of this study is to prove the feasibility of providing virtual environments controlled through surface electromyography that can be used for the future training of people using prosthetics or with upper limb motor impairments. We propose that simple control algorithms can be a more natural and robust way to interact with prostheses and assistive robotics in general than complex multipurpose machine learning approaches. Additionally, we discuss the advantages and disadvantages of adding intelligence to the setup to automatically assist grasping activities. The results show very good performance across all participants who share similar opinions regarding the execution of each of the proposed control modes.

## 1. Introduction

An amputation is the removal by surgery of a body part because of injury or disease. Most amputations caused by diabetes [1] usually affect the lower limb, while upper limb amputations are mainly due to traumatic events produced by a variety of causes. In 2017, between 50 and 60 million people worldwide where living with limb amputation due to a traumatic cause, where falls (36.2%), road injuries (15.7%), other transportation injuries (11.2%), and mechanical forces (10.4%) were the most common events leading to this condition [2]. For the upper limb, amputation is particularly disabling as most of the activities of daily living (ADLs) rely on the use of the hand and the arm to manipulate elements of the environment.

The rapid development of prosthetics has provided a way of substituting the lost limb not only aesthetically but functionally. In recent years, a variety of actuated upper limb prostheses have been developed to provide end users with the capability to grasp and manipulate objects during their ADLs. One of the most natural ways of interfacing the prosthesis is myoelectric control [3]. This type of control allows decoding motor intentions from the residual electrical activity recorded on the stump, which is the area of a limb that has been amputated. This technique involves the use of surface or intramuscular electrodes [4]. Commercial algorithms usually rely on simple and classical approaches, such as ON/OFF. This technique consists of defining numerical thresholds and checking whether the amplitude of the muscle contraction exceeds this value to activate (ON) or deactivate (OFF) a certain movement. This technique is commonly applied to decode wrist movements or the opening and closing of the hand [5,6]. Although the last decade of research has been actively working on the decoding of more complex activities, e.g., using pattern recognition to extract individual finger movements [7] or different hand motion patterns [8], these algorithms are still far from achieving perfect accuracy, so end users may still prefer the use of simple and robust algorithms. In this regard, a recent study shows that direct control outperforms pattern recognition in upper limb prosthetic control [9].

Nevertheless, myoelectric control is not always as easy to manage as other interaction methods. End users need to train subject-specific control algorithms to master a reliable and natural interaction with the prosthesis. For this purpose, there are several physical training systems that can be used to improve performance. One classical example is the Box and Block Test [10]. This test, initially used to measure dexterity in upper limb motor disability when grasping objects from side to side, is commonly used to train prosthetics control. A similar approach is the Clothespin Relocation Test [11] or multitask setups such as the Action Research Arm Test, very often used in stroke assessment [12]. The main drawback of these systems is that end users need the physical prosthesis with its associated costs before being sure of its suitability for their particular situation.

To avoid unnecessary expenses and frustration for end users, an excellent way of testing and training a myoelectric control approach is virtualisation. Virtual reality provides the possibility of creating a great variety of environments, including the aforementioned conventional tests, to allow the evaluation of any myoelectric control algorithm without the need for the actual prosthesis. This concept has been tested in the classical Box and Block Test [13] or combined with vibrotactile somatosensory stimulation [14]. More recently, augmented reality has also been used for this purpose, for example in combination with visual interaction [15] or using electromyograpy (EMG) to tune robot forces [16].

In this paper, we propose a daily living situation where objects in a kitchen can be grasped and stored in specific containers using a virtual robot arm. Similar environments have been previously tested with different purposes [17,18] but using complex machine learning algorithms. The main goal of this study is to prove the feasibility of providing virtual environments controlled through surface electromyography that can be used for future training of people using prosthetics or with upper limb motor impairments. We analyse the benefits of using simple ON/OFF algorithms in combination with state machines to easily accomplish grasping tasks in a natural and intuitive way. We also evaluate the advantage of adding a certain amount of automation to the task.

## 2. Methods

### 2.1. Virtual Environment

The proposed virtual environment consists of several daily living objects on a table, a box for placing the objects and an assistive robot arm to interact with them in real time using several control modes. Therefore the users, depending on their control actions, can perform pick and place tasks. We describe next the details of the virtual environment and the tools used to create it.

#### 2.1.1. Virtual Reality Software

The software used for the development of the virtual environment is Unity. Unity is a video game engine with programming routines to design, create and run a video game. Furthermore, the software is available on several platforms. Unity provides a tridimensional scene of the assistive environment and allows controlling the behavior of the robot arm, so that the user can interact with the objects in the scene.

#### 2.1.2. Appearance of the Virtual Environment

**Figure 1:**
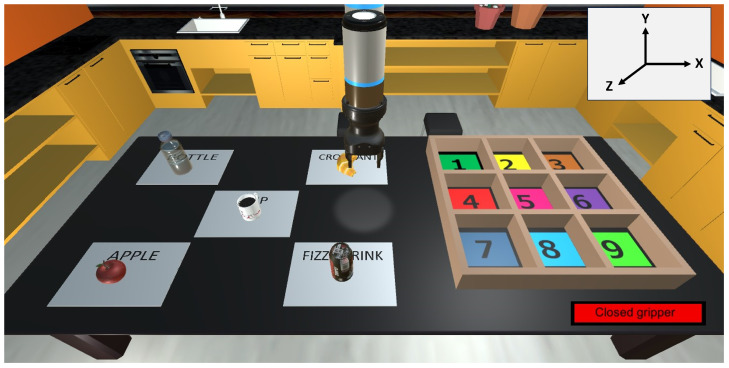
Virtual environment.

The designed virtual scene has the appearance of a kitchen (Figure 1). The purpose of using this scene is to provide the user with a familiar environment. Therefore, the feeling of immersion in the virtual environment is increased.

The main element of the scene is the robot used for the grasping actions. This robot is simulated with a Cartesian approach allowing 3D movements (X, Y, and Z axis) on the scene. To execute these movements the robot’s joints are not used. Instead, three Empty Objects have been created in Unity, to which the Articulation Body component has been added. Each of these components is configured as a prismatic joint so that the movement can only be performed in a straight line along one axis. Furthermore, this movement is constrained so that the robot cannot move outside the limits of the table, and thus outside the limits of what the user sees during the test.

**Figure 2:**
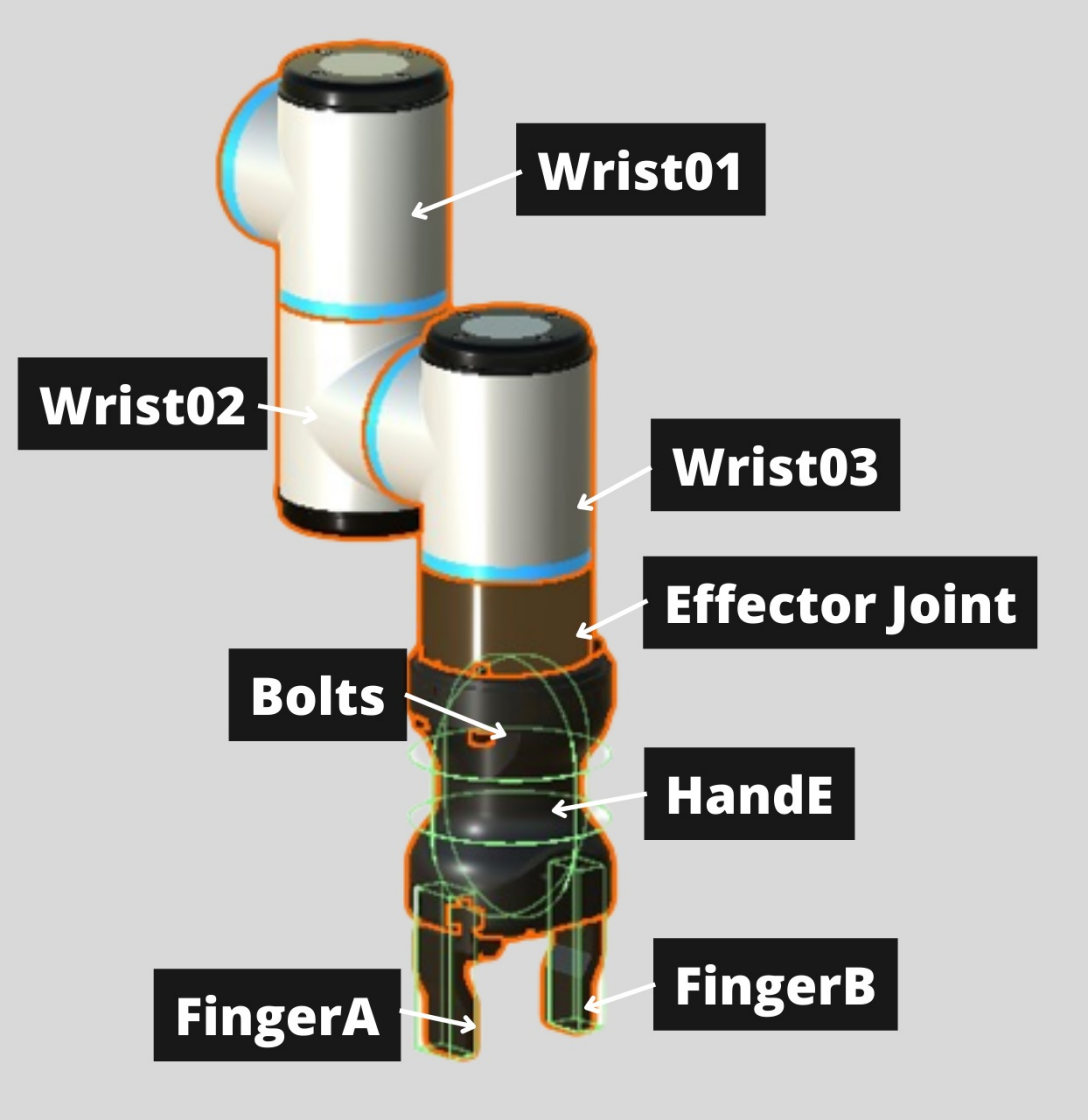
Robot elements.

The elements of the robot are shown in Figure 2. The end effector used in this simulation for grasping objects is the Robotiq Hand-E Gripper. The elements FingerA and FingerB correspond to each of the grippers that open and close to grasp the objects. A prismatic joint has been added to each of them. The movement of these joints is limited so that the grippers do not open beyond the width of the robot. The user must position the robot so that the object is positioned between the grippers. Once in this position, control actions close both grippers and the collision between the grippers and the object allows the object to move.

**Figure 3:**
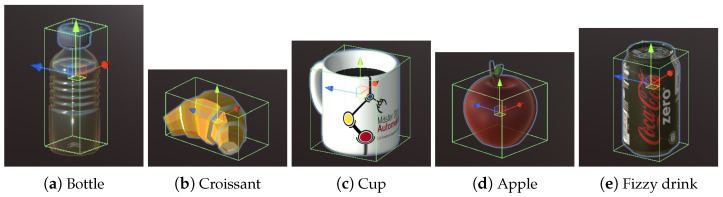
Objects of the environment.

The scene also includes objects that are manipulated by the user by commanding the robotic arm (Figure 3). These objects are a bottle, a croissant, a cup, an apple, and a fizzy drink; these have been chosen because they are common elements in a kitchen and familiar to the users. All objects are affected by gravity and collide with other objects (including the gripper) when they are in contact. Thus, the robot is able to grasp them in a realistic way. The objects to be moved are surrounded by a green rectangular prism which corresponds to a Box Collider. This element allows the robot to collide with the object and grip it, this box shape makes gripping easier. All objects are assumed rigid to avoid gripping issues, as the goal is to test myoelectric control but not realistic physics.

There is also a box in which the objects must be placed during the tests. This box has nine different positions, which have been numbered and identified with different colors. The remaining elements include the walls, the floor, the table and the kitchen furniture; and are added to provide a more pleasant environment for the user.

#### 2.1.3. Control Modes

**Figure 4:**
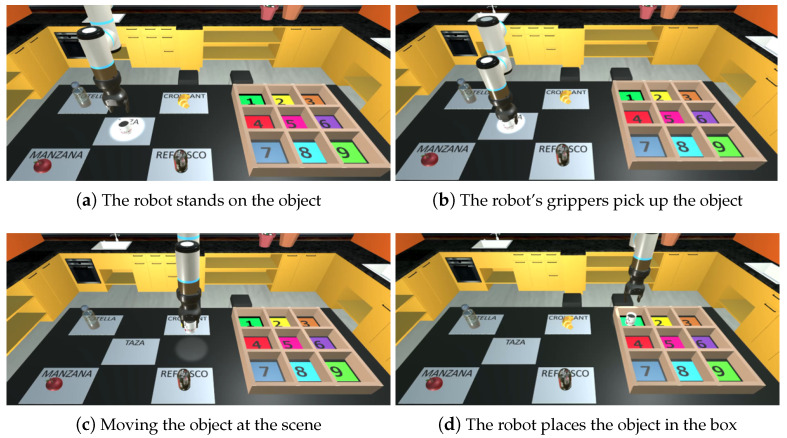
Robot movement in the scene during pick and place.

The proposed application has three different control modes. In each of these modes the movements of the robotic arm are controlled differently:

- Mode 1: Free motion on three axes and manual grasping. The user is in charge of most of the control of the robot. In this mode the user can perform eight different actions. These actions are the movement of the end effector of the robot, both positive and negative, in all three Cartesian axes, and the opening and closing of the gripper.
- Mode 2: Free motion on two axes and automatic grasping. The number of actions that the user can perform is reduced to six. The movements that the robot can perform are the translations in the *x* and *z* axes, both in the positive and negative directions. The other two user actions command the robot to automatically grasp or place an object. Figure 4 illustrates the robot’s movement in the scene. The robot positions itself on the closest object, grasps it with its grippers, and transports it to the target position. Upon reaching the desired box position, the robot deposits the object.
- Mode 3: Automatic motion and grasping. This is the only mode in which all the robot’s movements are performed automatically. The user chooses, by selecting the options implemented in the virtual interface, the object to grasp and the position where the object will be placed.

### 2.2. Myoelectric Control

To interact with the virtual environment described above, a myoelectric control approach has been implemented. In the following subsections, we describe how EMG signals are acquired, processed, and classified.

#### 2.2.1. Selected Muscles and Movements

This application seeks to replicate states or movements of the human hand that allow the manipulation of objects: wrist flexion, wrist extension, co-contraction (concurrent flexion and extension) and rest. Flexion and extension are two antagonist movements. The first is the inclination of the palm of the hand towards the front of the forearm. The second is the movement in the opposite direction. Co-contraction consists of a simultaneous activation of flexion and extension. The simplest way to perform this movement is to close the hand and exert force as if an object is being grasped. The chosen muscular inputs are intended to be useful for patients with transradial amputation. This type of amputation is produced below the elbow. The muscles that are located in this area and are involved in the movements mentioned above are Brachioradialis and Flexor Carpi Radialis. For this reason two bipolar surface EMG electrodes are placed on the belly of these muscles, longitudinal to the muscle fiber.

#### 2.2.2. Signal Processing

The Noraxon Mini DTS receiver is used for the acquisition of EMG signals during muscle contractions. The sensors used are bipolar electrodes model 548 with a 16-bit resolution and allow a sampling rate of 1500 Hz or 3000 Hz. The Mini DTS receiver collects the EMG signals from the sensors via radio. This information is transmitted to Noraxon’s own software (Noraxon MR3) via a USB connection.

The parameters necessary for the acquisition, conditioning and processing of the EMG signal are tuned in the Noraxon software. The typical range of EMG signals is from 10 to 500 Hz although they can reach higher values. The chosen sampling frequency is 1500 Hz. A low-pass filter of 500 Hz is also set to limit all interference in the signal caused by high frequencies. The last step of signal conditioning is full wave rectification of the signal and finally smoothing to remove unwanted noise and obtain the linear envelope.

For communication between Matlab and Noraxon, an HTTP application layer protocol is used. In this case, Noraxon MR3 is the server and Matlab is the client. Matlab acquires the readings of the EMG signals through HTTP streaming socket requests.

#### 2.2.3. Control Approach

**Figure 5:**
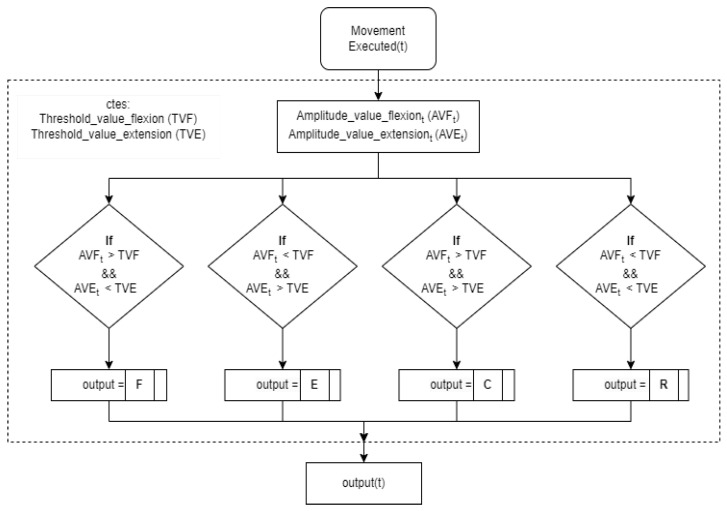
Scheme of the algorithm for the classification of movements.

In this application a mixed myoelectric control approach between ON/OFF control and a state machine is used. The ON/OFF control mode (Figure 5) is used to classify which type of movement is being detected by the user (flexion, extension, co-contraction or rest). Two thresholds are defined, one for extension and one for flexion movement (TVF and TVE). When the amplitude of the user’s EMG signal ($AVF_{t}$ and $AVE_{t}$) exceeds one of these thresholds, flexion or extension movement is detected. If both thresholds are exceeded, a co-contraction movement is detected. The state machine, which depends on the classification of movements by the ON/OFF control mode ($output_{t}$) mentioned above, is used to define the robot’s states as well as the transition between them. The thresholds that are established for the ON/OFF control mode are customised for each user. To have a more intuitive interaction, flexion movements are related to movements in the negative axis of the robot and extension movements to the positive axis of the robot. The co-contraction movements allow the transition between the states of the state machine. Each mode deals with the muscular inputs in a different way.

**Figure 6:**
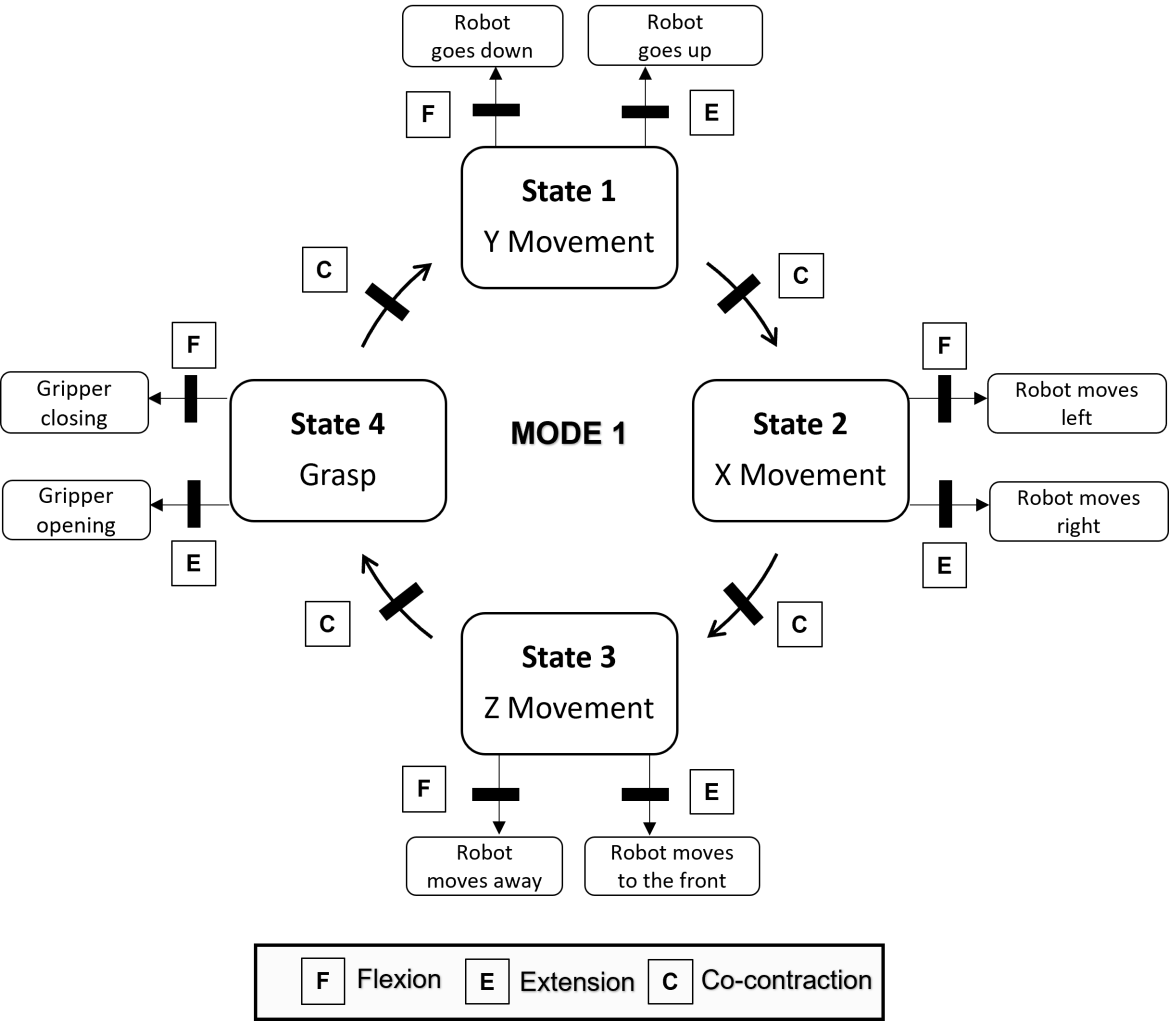
State machine of Mode 1.

**Figure 7:**
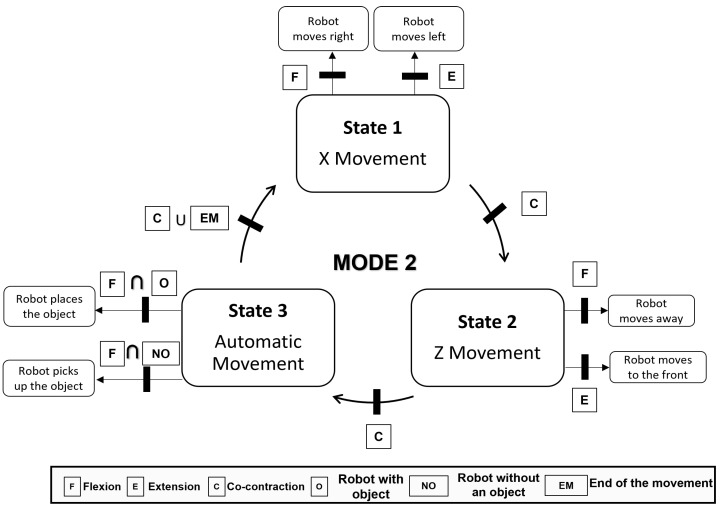
State machine of Mode 2.

**Figure 8:**
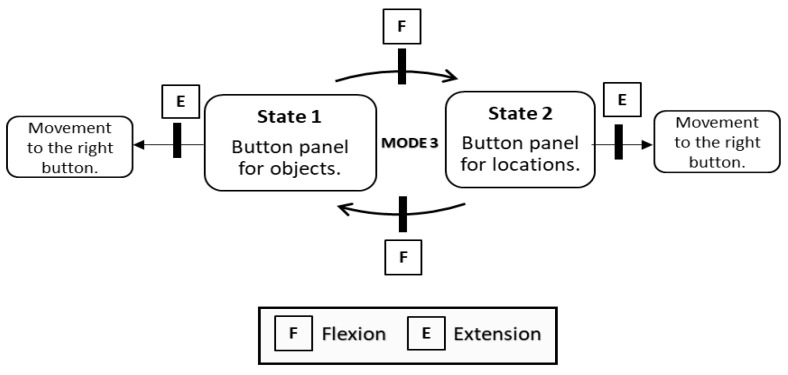
State machine of Mode 3.

- In mode 1, the state machine has 4 states. This mode provides the user with the highest number of degrees of freedom. Figure 6 shows the distribution of these states and how each movement is associated with a replica of the robot’s movement in the virtual environment.
- Mode 2 has one state less because the user is not provided with the ability to manipulate the object in the y-axis (Figure 7). The robot automatically performs this function as already mentioned in the virtual environment section.
- Mode 3 significantly differs from the previous modes. The user performs extension and flexion movements to move around a button panel and selects one of the options (Figure 8). In this mode there are two states, the first in which the user is selecting the object and the second when the object’s destination is selected. The transition between states is done automatically when the robot finishes the pick-up or drop-off function. For this reason, the co-contraction movement has no functionality in this mode.

#### 2.2.4. Biofeedback Interface

**Figure 9:**
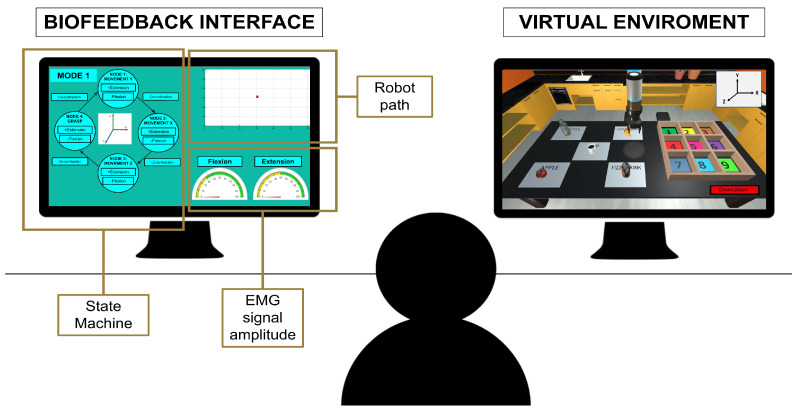
Representation of the user’s visual feedback during the experiment. On the left side the visual biofeedback and on the right side the virtual environment.

To provide the user with information about their muscular activity and the control performance, an interface (Figure 9) with several graphical elements has been developed using Matlab tools.

The first element is a schematic representing the state machine. The user can follow in real time which state is activated and which movement (flexion or extension) is performing.

The second element is a figure with two speedometers showing the amplitude of the flexion and extension EMG signal that the user is reaching. In addition, these speedometers are customised according to the thresholds of each user: a green band indicates the area where movement is detected and a yellow band denotes no detection.

In mode 1, unlike the other two modes, a graph is added to represent the robot’s trajectory through the virtual environment. This element is provided to track if all users follow the same strategy of movements required to pick up and drop off the object. In mode 2 and mode 3 this element does not appear because the user’s movements are more limited and therefore the strategy is very similar.

### 2.3. Experimental Protocol

8 users (3 female and 5 male, with ages 24.75 ± 4.65) participated in the experiment. All of them signed the corresponding informed consent and had no history of neurological or physical disorders affecting the instrumented arm. A total of 75% of the participants had prior experience with virtual environments. In contrast, only 37.5% of the subjects had previous experience with EMG signals for myoelectric control. The experiment consisted on the repetition of 5 pick and place activities (1 for each object) using all three implemented modes. Users were allowed to rest between repetitions and a limited number of errors was allowed if the task was finished. If users were not able to finish, that repetition was labelled as a failure.

## 3. Results

This section provides results for all the proposed performance metrics and the answers to the participants survey.

**Figure 10:**
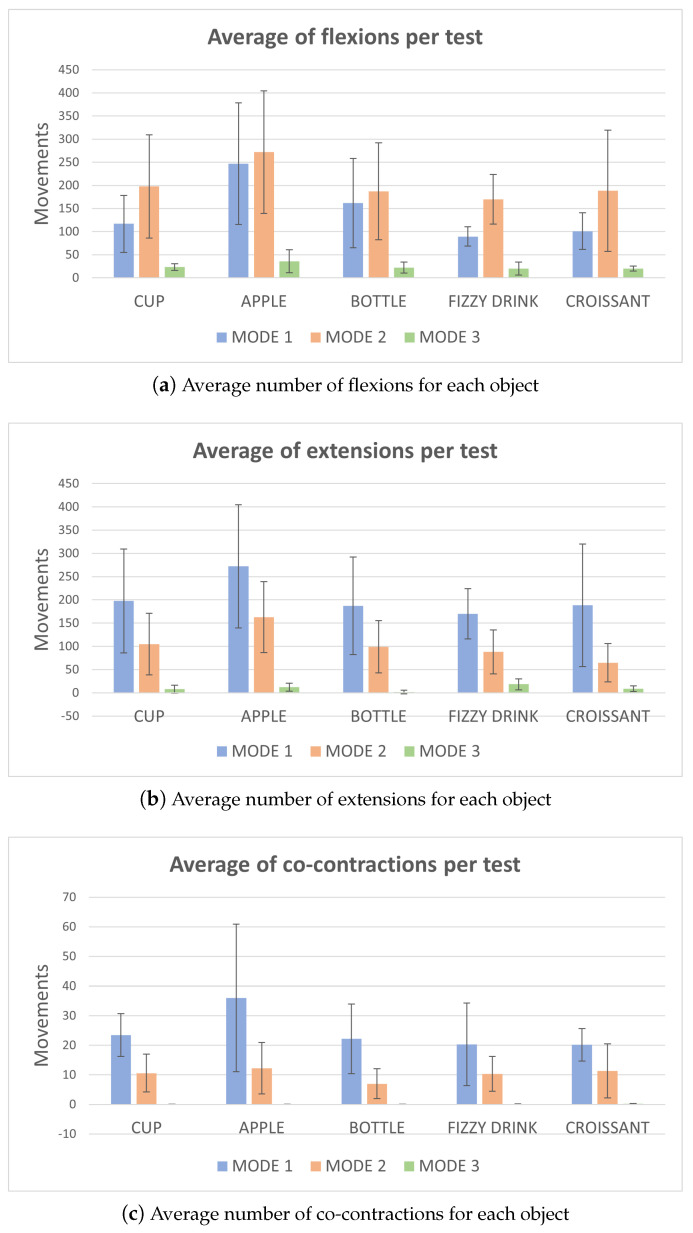
Analysis of number of movements.

As can be seen in Figure 10, the control mode that requires the greatest number of contractions is mode 1. This is due to the fact that it is the mode that allows a higher number of degrees of freedom. Also for this reason, mode 3 is the one that requires less contractions as it only needs a few movements to select the final option. It has been determined that the number of movements required is influenced by the degrees of freedom of each mode. The greater the number of degrees of freedom, the greater the number of movements required. Therefore, the more automatic the mode, the faster the user can access the object. The object that requires the greatest number of movements is the apple, because it is the furthest object from the initial position and the most difficult one to manipulate due to its shape. On the contrary, objects such as the fizzy drink or the croissant imply a smaller number of contractions in general.

When analysing individual performance (Table 1, Table 2 and Table 3) it can be observed that numbers are very variable across subjects, having participants 1 and 2 a very good performance, while other participants obtain worse results (for instance User 3 in mode 1 or User 4 in mode 2). However, mode 3 has a very good general performance (expected in some particular cases).

**Figure 11:**
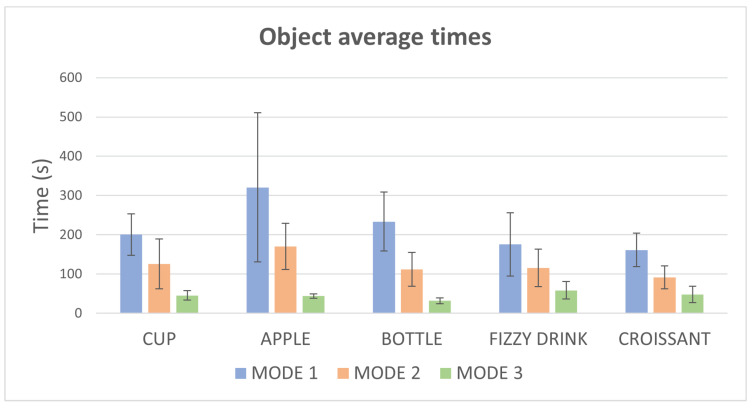
Graph of average times for each object in different modes.

A similar result is found for the average time per test (Figure 11). Mode 1 is the slowest mode while mode 3 is clearly the fastest way of interacting with the virtual environment. Again, the apple is the object that takes the longest time while the croissant and the fizzy drink imply shortest grasping times.

Table 4 shows the values of the total time taken by each subject to perform the whole test. The different threshold values for flexion and extension set for each user can also be observed. Based on these values it is important to note that all users finished the test in less than the maximum time (90 min). The minimum time is always above 40 min, which is not excessive as it covers 15 different tests. It is also remarkable the importance of customising the thresholds as none of the users repeat values, although most of them are in the range of 20 to 60 μV. Regarding muscle fatigue and usage the thresholds set in flexion are lower than those in extension. This indicates that the muscle Flexor Carpi Radialis is generally less exercised than muscle Brachioradialis. 

### Survey Results

Participants were asked to answer a survey with 4 different sections: 1—Participant Background, 2—Myoelectric Control, 3—Virtual Environment, and 4—General Opinion.

The answers to the survey (Section 1) show that most of them are right-handed (75%) and have high-level education. They were generally fit and most of them did not know anyone with physical amputations (87.5%).

In relation to the questions on Section 2 of the survey (myoelectric control), the users gave an average score of 8.25 on the comfort of the sensors and 8.5 on the simplicity of learning myoelectric control. The Matlab interface is rated 9.4 and users have suggested improvements such as acoustic or vibrotactile feedback. The average degree of muscular fatigue of the users is 6 and the average degree of mental fatigue is 3. Finally, the movement that was most difficult for 75% of the users was the co-contraction (4.5/10).

Regarding Section 3 (virtual environment), a total of 87.5% of participants think that mode 1 is the most difficult to control, followed by mode 2. There is total agreement that mode 3 is the easiest. The average difficulty rated by users for each mode is: Mode 1, 6.75; Mode 2, 3.75; and Mode 3, 2.38. The final rating of the most difficult object to be manipulated in mode 1 was the bottle of water with 62.5%, followed by the apple and the fizzy drink. In the opposite case, the easiest object to handle was the croissant with 37.5% and the apple with the same percentage followed by the fizzy drink. The user’s opinion on the ease or difficulty is given by how the geometry of the object is adapted to the gripper. The arrangement of the object on the table also influences this, as for example the bottle is more likely to fall off the table after a wrong movement. All users considered mode 2 navigation more advantageous and comfortable compared to mode 1 and mode 3. The participants consider that mode 2 with 75% is the one that can be best applied in a daily habit followed by mode 3.

Finally, for Section 4, the final score given by users of this application is 9.25/10. Indeed, 100% of the subjects consider it to be a good method of controlling assistive technology, and 75% of users consider it to be a reliable system.

## 4. Discussion

This study shows the development of a virtual system by which people with upper limb amputation can train myoelectric control simulating the performance of ADLs such as handling and grasping objects. This framework provides different control modes, variability of elements to grasp, possibility of changing grippers and different assessment metrics.

The system’s performance has been evaluated through a series of tests. A total of 120 tests have been carried out. A hundred and ten tests were successfully completed, which represents 92% of the total. Uncompleted tests were usually not caused by an under-performance of the myoelectric control but because of misplacement of objects that made them physically not graspable, i.e., objects falling in a strange position. Only in three tests were some users unable to complete the tests due to mental and muscular fatigue. With a success rate of 92% and an average time below the maximum time originally planned, we can say that the results of the evaluation of this myoelectric control are very satisfactory. According to previous studies, age may influence the acceptance of this kind of interaction as it has been proven to affect the use of prosthetics in general [19]. For this feasibility test, a specific healthy population has been chosen but future assessment should be carried out to infer population differences.

Participants have different experiences of the grasping of the same object depending on the control mode. This is reflected in the time they spent, as well as in the survey answers. The mode in which the users required more time was mode 1. This mode is also the one that users evaluated as the most complex, arguing that too many state changes were required to complete a task. The mode in which users needed less time to perform a task was mode 3. Furthermore, this is the mode that was selected in the survey as the easiest to control. Despite this, most users chose mode 2 as the mode they would use to perform ADLs. This is due to the fact that mode 2 offers a greater freedom of movement compared to mode 3, with a little assistance compared to mode 1.

The choice of the type of myoelectric control plays an important role when interfacing robots. Despite the fact that more complex algorithms have been used in other studies [7,8], in this study a simple ON/OFF algorithm combined with state machine control was chosen. The combined use of myoelectric ON/OFF control and finite state machine aws very successful and very intuitive to all participants. Furthermore, in this application we introduce three degrees of freedom in the definition of the state machine compared to other applications designed using only hand flexion and extension [20,21]. It is worth mentioning that very complex setups are difficult to control with any myoelectric control algorithm, even with a higher number of outputs and advanced artificial intelligence [22,23]. Common prosthetics are usually very simple, often just for grasping, as it is the main functionality missing in amputees.

This underscores that the complexity of algorithms does not necessarily translate into better precision or performance in prosthetic control, particularly considering the significant data requirements needed to improve complex models. The latter requires increasing both the time and the number of sessions per user [24].

The activity chosen for myoelectric control is similar to the conventional Box and Block Test. Previous studies have shown how carrying out this test in a virtual reality environment improves user control skills [13]. In this previous study, shorter times were achieved than those of the current study. However, this is not a disadvantage since the purpose of this study is to test the feasibility of the system in which users perform the activities successfully regardless of the time used. Additionally, it also includes a higher variability than the Box and Block Test as different shapes of objects are present in the virtual environment and placing positions are variable. Future testing will evaluate how training reduces time and improves accuracy in grasping. In previous studies, participants have performed similar myoelectric training [25], but in those cases the user only had to interact with the environment according to a single mode. In this study, the users can try different ways to interact with the assistive robot, so that they have the opportunity to choose the one that best suits their needs.

The future aim of this framework is for users to learn how to effectively control an assistive robot, i.e., train themselves in myoelectric interaction. In other studies, this interaction was carried out with real assistive robots such as prosthetic hands [7]. In this study, a robot was used in a virtual environment. The main advantage of virtuality is that it provides a custom environment previous to the actual use of a real robot that allows the user to test the feasibility of myoelectric control and reduces the costs of acquiring those robotic systems in advance. In this case, the proposed framework can be used both to train myoelectric control for prosthetics or for interaction with assistive robots. In this sense, the proposed study will also be tested in the future with an actual assistive robot in a real environment. Another possible upgrade is the introduction of immersive virtual reality by using a headset. This will enhance the feeling of reality for the end user and provide a way of training in a digital twin version of real daily living contexts.

The proposed virtual platform is not limited to the manipulation of the gripper used in the obtained results, but it can also be adapted to interact with other types of robotic grippers. For instance, rigid-link grippers [26] offer precise control but may lack adaptability to irregular shapes, whereas compliant underactuated grippers [27] are more flexible but require more complex control. By integrating accurate models of these grippers into the virtual environment and developing adaptive control algorithms, users can explore and familiarise themselves with the characteristics of each gripper type. This expands the scope of the platform to address various applications and provides opportunities for research in the field of robotic manipulation.

## 5. Conclusions

Most of the activities of daily life involve the manipulation and grasping of objects. In this paper, we present a virtual environment of a daily living situation controlled through surface electromyography that will be tested in the future for the training of people using prosthetics or with upper limb motor impairments. The control used to direct the robot’s movements is a myoelectric signal control. This virtual framework was set up as a domestic environment in order to increase the user’s sense of immersion. In this environment, there is an assistive robotic arm that has four degrees of freedom, due to the movement of three prismatic joints and the grip of the gripper on its end effector, and there are different objects that the user has the possibility to manipulate. In addition, different modes were implemented in order to obtain different ways of controlling the robot, each of which has a different degree of autonomy on the part of the robot. Finally, in order to evaluate the performance of the myoelectric control system in the virtual environment, tests were carried out with different users in which different parameters were analysed, as well as the experiences of the subjects. The success rate obtained in these trials shows how the tests were mostly completed, proving the feasibility of the system.

## Figures and Tables

**Table 1:** Number of extensions, flexions, and co-contractions in each test in mode 1.

USERS	MODE 1														
	Bottle			Croissant			Cup			Apple			Fizzy Drink		
	Flex	Ext	Co	Flex	Ext	Co	Flex	Ext	Co	Flex	Ext	Co	Flex	Ext	Co
User 1	73	88	43	34	40	23	29	33	19	50	52	19	68	85	51
User 2	-	-	-	139	132	27	80	130	23	98	125	11	57	100	15
User 3	-	-	-	86	461	23	175	285	27	450	490	87	90	203	11
User 4	210	282	15	154	207	23	162	266	15	261	314	23	104	207	15
User 5	43	59	19	83	131	15	40	42	23	234	296	31	118	191	19
User 6	252	246	19	97	168	11	148	248	15	276	313	31	98	190	11
User 7	-	-	-	-	-	-	112	285	35	228	312	27	90	214	20
User 8	231	260	15	114	179	19	189	293	31	379	274	59	-	-	-

**Table 2:** Number of extensions, flexions, and co-contractions in each test in mode 2.

USERS	MODE 2														
	Bottle			Croissant			Cup			Apple			Fizzy Drink		
	Flex	Ext	Co	Flex	Ext	Co	Flex	Ext	Co	Flex	Ext	Co	Flex	Ext	Co
User 1	21	20	4	18	18	14	15	17	7	34	48	7	22	28	7
User 2	42	50	4	19	32	4	37	50	4	42	51	7	34	71	8
User 3	106	129	7	30	77	4	-	-	-	140	171	11	64	87	7
User 4	129	141	4	29	98	13	-	-	-	187	237	17	86	117	10
User 5	31	27	7	54	6	7	95	147	10	155	179	7	10	14	4
User 6	111	135	4	25	89	7	96	155	11	139	158	14	77	146	10
User 7	92	137	7	15	122	32	-	-	-	126	246	31	78	122	23
User 8	131	153	19	26	77	10	108	155	2	146	211	4	91	120	14

**Table 3:** Number of extensions, flexions, and co-contractions in each test in mode 3.

USERS	MODE 3														
	Bottle			Croissant			Cup			Apple			Fizzy Drink		
	Flex	Ext	Co	Flex	Ext	Co	Flex	Ext	Co	Flex	Ext	Co	Flex	Ext	Co
User 1	2	0	0	8	13	2	4	4	0	2	6	0	6	13	0
User 2	5	2	0	3	7	0	3	4	0	3	30	0	2	10	0
User 3	2	0	0	2	2	0	6	5	0	2	6	0	2	13	0
User 4	11	0	0	2	4	0	3	8	0	2	19	0	4	12	0
User 5	11	0	0	4	8	0	5	4	0	5	11	0	9	34	0
User 6	3	0	0	3	2	0	2	4	0	3	7	0	3	8	0
User 7	3	0	0	2	17	0	3	29	0	3	7	0	3	40	1
User 8	4	11	0	3	17	0	2	4	0	3	11	0	2	18	0

**Table 4:** Total test time values, flexion and extension thresholds per user, success rate per user. The total test time includes the pauses between the different tests and the times of failed tests.

Users	Total Test Time (min)	Flexion Threshold (µV)	Extension Threshold (µV)	SUCCESS Rate (%)
User 1	58′01″	20	30	100
User 2	45′50″	40	50	93.33
User 3	58′56″	40	60	86.67
User 4	50′04″	70	60	93.33
User 5	50′45″	60	100	100
User 6	42′07″	30	60	100
User 7	51′	20	30	80
User 8	57′	30	50	93.33

## Data Availability

Data is available under request to the corresponding author.
